# The Prevalence of Visual Impairment and Refractive Errors among a Youth Population in Mozambique: Evidence of the Need for Intervention

**DOI:** 10.3390/children8100892

**Published:** 2021-10-06

**Authors:** Dulnério B. Sengo, Isaura I. D. B. Dos Santos, Momade F. Faquihe, Hermenegildo B. J. F. Tomo, Alcino M. Muaprato, Sualé Puchar, Guida M. R. J. Lôbo, Inmaculada López-Izquierdo, Pablo Caballero

**Affiliations:** 1 Faculty of Health Sciences, Lúrio University, Nampula City 3100, Mozambique; dulnerio@yahoo.com.br (D.B.S.); ibrito@unilurio.ac.mz (I.I.D.B.D.S.); mfaquihi@unilurio.ac.mz (M.F.F.); Htomo@unilurio.ac.mz (H.B.J.F.T.); alcinomuaprato87@gmail.com (A.M.M.); spuchar@unilurio.ac.mz (S.P.); guidademiranda@gmail.com (G.M.R.J.L.); 2 Department of Physics of Condensed Matter, Optics Area, University of Seville, Reina Mercedes St., 41012 Seville, Spain; 3 Department of Community Nursing, Preventive Medicine and Public Health and History of Science, University of Alicante, 03690 Alicante, Spain; pablo.caballero@ua.es

**Keywords:** visual impairment, refractive error, schoolchildren, Mozambique

## Abstract

Visual impairment (VI) can significantly interfere in the child’s daily activities and quality of life, having a negative effect on their development and learning. The aim of the study was to determine the prevalence of VI and associated demographic factors in students examined during the program “Moçambique te vejo melhor”. This study was cross-sectional and retrospective, based on the 2018/19 edition of the program. Eye examinations were performed in secondary school students, aged between 12 and 20 years, of five districts in Nampula province. The examination included visual acuity, non-cycloplegic refraction and assessment of the anterior and posterior segment and ocular adnexa. The prevalence of uncorrected, presenting and best-corrected VI found was 18.3%, 10.8%, and 5.0%, respectively. Refractive error (RE) had a prevalence of 24.7%, and the age groups between 15–17 years and 18–20 years were significantly associated with myopia (with OR: 4.9 and OR: 8.8, respectively), as well as the 11th and 12th grade (OR: 8.1 and OR: 10.7, respectively), and Malema district had association with myopia (ORa: 0.4) and hyperopia (ORa: 0.4 and OR: 0.3) as a protective factor. The prevalence of RE and VI was relatively high, showing the need for greater intervention at the school level.

## 1. Introduction

The visual impairment (VI) results when an eye condition affects the visual system and one or more of its vision functions, such a visual acuity, can interfere significantly with daily activities and the individual’s quality of life. VI can have a negative effect on development and learning for children, especially when it occurs during the first years of life. More than 85% of a child’s learning is through visual stimuli [1,2].

Globally, it is estimated that 19 million children are blind or visually impaired, most of which is treatable or preventable, with a higher prevalence in low-income countries (9/10,000 children) compared to middle and high-income countries (7/10,000 and 4/10,000, respectively) [3].

Refractive errors (REs), eye disorders that occurs when the eye cannot clearly focus the images from the outside world, resulting from a mismatch between the axial length of the eye and its optical power [4,5], have been the main cause of VI in children, and they are the most prevalent visual disorder among children, affecting more than 20% [6]. REs have been associated with genetic and environmental factors, such as the influence of the gene loci (AREG, GABRR1, and PDE10A), near work distances and time spent in outdoor activities [7,8].

However, in Mozambique, there are no published studies on the prevalence and causes of VI in children. Data are needed to better plan strategies and policies in order to combat preventable VI in this group, thus reducing the high costs for the health system and society [1,7].

In developing countries, where barriers to accessing eye health services prevail, screening programs in schools have the potential to promote eye health and the early detection of eye diseases and prevention of VI, particularly for children living in remote locations [3].

The program “Moçambique te vejo melhor” (“Mozambique see you better”), is developed by Universidade Lúrio, with the aim of promoting eye health in schools located in remote regions in Nampula and surrounding districts. Vision Screening has been one of the adopted mechanisms in the program, aiming to assess the eye health status and detect eye problems in students. This program has taken place during the month of September of each year and covers secondary level schools [9]. Therefore, this study aims to determine the prevalence of cases of VI and associated demographic factors in examined students during the program. The results obtained will provide important information for the improvement of the program and extension to other territories in the country.

## 2. Materials and Methods

**Figure A1:**
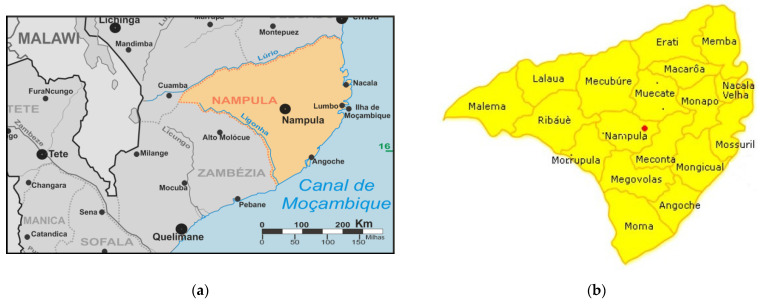
(**a**) Location of Nampula within Mozambique (**b**) Distribution of the districts of Nampula.

This was a cross-sectional and retrospective study, based on clinical records of screenings carried out during the program “Moçambique te vejo melhor” 2018 and 2019 edition. Public schools from five districts (Mogovolas, Malema, Rapale, Meconta, and Mossuril) in Nampula province were part of the program. Nampula is located in the northeast of Mozambique, 2036 km (by road) from Maputo (capital of the country), with an area of 81,606 km^2^. It is limited to the north by the provinces of Cabo Delgado and Niassa, to the south by the province of Zambézia, and to the east by the Indian Ocean (Appendix A Figure A1) [10].

Nampula is the most populous province in Mozambique, with 20.6% (5,758,920 inhabitants) of the general population, with the district of Mogovolas having 415,407 inhabitants, Malema with 223,791, Rapale with 174,707, Meconta with 250,425 and Mossuril with 142,787 inhabitants. The province has 3,002,676 children (0–17 years), where 934,543 (31.1%) live in urban areas and 2,068,133 (68.9%) in rural areas [11]. The “Moçambique te vejo melhor” program has been carried out in coordination with the district education directorates since 2014, with the aim promote the eye health of secondary school students (8th, 9th, 10th, 11th, and 12th grade) [9]. Currently, schooling in Mozambique is free and compulsory from the 1st to the 9th grade (from 6 to 15 years).

Ethical aspects: The study was previously approved by the Institutional Committee of Bioethics for Health of Lúrio University (CIBSUL), on 17 December 2019, with ref: 51/Dec/CBISUL/19. During the program, students were examined through Signed Informed Consent (by parents and students aged ≥18 years) and Assent (by students aged <18 years) written in Portuguese.

Clinical procedures: Clinical examinations were performed by a group of eight optometrists with more than five years of clinical experience each one. All cases of ocular pathologies and VI that did not improve with better correction were referred to the university clinic for complementary exams and identification of the cause. During the screening, socio-demographic data (district, academic level, age, gender) of the students were collected. To assess visual acuity (VA) logarithm tables of the minimum angle of resolution (logMAR) were used, at 4 m, monocularly, and the value recorded on the logMAR scale. The objective refraction was static (with a Keeller Professional Streak Retinoscope) without cycloplegic, and then the subjective refraction was performed. Due to the impossibility of using a cycloplegic, the use of an autorefractor was discarded as it could overestimate the prevalence of myopia in students with very active accommodation [12]. To evaluate the ocular structures, anterior and posterior segments, a point flashlight, magnifying loupe and a professional Keeler ophthalmoscope (for direct ophthalmoscopy) were used.

Inclusion and exclusion criteria: Clinical record that were duly completed and with the informed consent and assent forms signed were included in the study.

Variable definitions: The refractive state definition was based on the spherical equivalent (SE = sphere + half of the cylinder value) of the subjective refraction [6,7], and myopia was considered for SE ≤ −0.50D, hyperopia for SE ≥ +2.00D, and emmetropia for SE > −0.50D and <+2.00D. Regarding to severity, it was considered mild for REs ≤ 3.00D, moderate between 3.25 and 6.00D, and high for >6.00D [7]. Astigmatism was considered (using negative cylinders) for values ≤ −0.75D. The type of astigmatism was defined according to the cylinder axis, as with-the-rule (WTR) for axes from 1° to 15° and from 165° to 180°, against-the-rule (ATR) for axes from 75° to 105°, and oblique (OBL) for axes from 16° to 74° and 106° to 164° [6]. Visual impairment (VI) and its respective category were defined based on the patient’s VA (logMAR scale). VI was considered for VA ≥ 0.30 (≤6/12 Snellen) [13], being mild for VA between 0.30 and 0.50 (6/12 and 6/18 Snellen), moderate >0.5 to 1.0 (<6/18 to 6/60 Snellen), severe >1.0 to 1.30 (<6/60 to 3/60 Snellen), and blindness >1.30 (<3/60 Snellen) [14]. “Uncorrected VI” was defined on the basis of uncorrected VA. While the “presenting VI” was according to the VA taken in the condition in which the patient presented himself for the exam, using his correction if any. “Best-corrected VI” was defined based on the VA taken after subjective refraction [15]. Cases of bilateral VI, the VA of the best eye was considered.

Statistical Analyses: A socio-demographic description of the sample was made, and the frequency of VI and its causes was determined. The correlation between the RE of the right and left eye was analyzed using Spearman’s correlation coefficient, and a statistical significance and high correlation was found (Rs: 0.85; *p* < 0.001), which is the reason that statistical analysis was made only with data from the right eye of each patient, as was done in similar studies [16]. For study the association between refractive error and socio-demographic characteristics (age, gender, school level, and district) the Odds Ratio and adjusted Odds Ratio (OR and ORa) was calculated by logistic and multiple regression with successive steps forward, with 95% confidence interval (CI) and 5% significance level. The adjustment was carried out using all available sociodemographic variables. All data analysis was performed using SPSS version 23.0 (SPSS Inc., Chicago, IL, USA). Statistical power [17] was calculated with the package WebPower of the language and environment for statistical computing R [18].

## 3. Results

In a total of 592 participants, 575 had the clinical records duly completed and with the respective signed informed consent form, as they took part in the study. The participants were aged between 12 and 20 years, with an average of 15.13 (±2.24) years. The majority (56.9%) of the participants were female. There were more students in the 8th grade (31.1%) followed by those in grade 9 (21.6%) in this study’s sample. Most participants were from Meconta (39.5%) and Mogovolas (25.7%) districts (Table 1).

The prevalence of uncorrected VI was 18.3%, The 18–20 age group had the highest prevalence (29.2%). Regarding to the school level, the prevalence was higher in the 11th and 12th grade (with 26.4% and 31.1%, respectively). The district of Meconta and Mossuril had the highest prevalence (both with 21.1%) (Table 1).

The prevalence of presenting VI was 10.8%, and the 18–20 age group had the highest prevalence (13.3%). As for the school level, the 11th grade had the highest prevalence (15.1%), and Malema had the highest prevalence among the districts involved (12.3%) (Table 1).

The prevalence of best-corrected VI was 5.0%, and the age group with the highest prevalence was 12–14 years (5.8%). Regarding to the school level, the 9th grade had the highest prevalence (6.5%). Rapale and Mossuril had the highest prevalence among districts (6.3%) (Table 1).

For the three types of VI, male gender had the highest prevalence and was mostly mild to moderate (Table 1).

Among patients with presenting VI, 53% achieved normal vision with the best correction.

Overall, refractive errors (REs) had a prevalence of 24.7%, mainly constituted by hyperopia (14.3%) to the detriment of myopia (10.4%). The age groups between 15–17 years and 18–20 years were significantly associated with the occurrence of myopia (with OR: 4.9 and ORa: 5.1, and OR: 8.8 and ORa: 12.5, respectively), with higher risk in the latter (Table 2).

The grade 11 and 12 had a significant association with the occurrence of myopia (OR: 8.1 and OR: 10.7, respectively). While Malema district had a significant association with myopia (ORa: 0.4) and hyperopia (ORa: 0.4 and OR: 0.3), however, as a protective factor for its occurrence (Table 2).

REs were mostly mild and moderate. Astigmatism had a prevalence of 8.3%, and it was mostly WTR with 6.4% (Table 3).

In 142 patients with RE, only 33.1% used correction. The prevalence of uncorrected refractive error (URE) was 16.5%.

The main cause of uncorrected VI was RE and amblyopia, with almost 40% resolved with the usual correction (day-to-day), and a little more than 70% resolved with the best correction. For the best-corrected VI, the main cause was amblyopia, followed by corneal opacity and congenital cataract, as the most prominent ones (Table 4).

## 4. Discussion

This study is the result of a visual health screening program promoted by Universidade Lúrio, which covers the remote areas of Nampula and surrounding districts. The research offers important preliminary results for the improvement of the program and extension to other regions of the country. The evidences on the magnitude and causes of VI are useful for monitoring progress and identifying priorities as part of the World Health Organization’s 2014–2019 global eye health action plan [19].

The prevalence of Uncorrected VI found in the study was 18.3%, similar to the study carried out in Vietnam [20] and Nepal–Kathmandu [21], which was 19.4% and 18.6%, respectively. However, in Ghana [22], the prevalence of Uncorrected VI was much lower (3.7%) than in other studies, which may be associated with the low proportion (3.2%) of myopia found in this study.

For presenting VI, the prevalence was 10.8%, similar to what was found in Nepal [21] and Malaysia [23], which was 9.1% and 10.1%, respectively. However, in Brazil [24] and Vietnam [20], the prevalence was slightly higher (with 14.5% and 12.2%, respectively), and in Ghana [22] it was much lower (3.7%). The prevalence of presenting VI is an indicator that so much reflects the coverage of the health system and accessibility to eye health services, which may vary depending on each country and region, and may also be determined by economic and socio-cultural factors that lead people to live with VI. In general, the most remote and poorest regions of low-income countries are the most disadvantaged, have less access to eye health services [25]. The unavailability of eye health services and geographic and financial limitations for their access can be pointed out as possible causes for a higher prevalence of presenting VI [26,27], since the five districts involved do not have optometrists or ophthalmologists for eye health services. The districts of Mogovolas, Malema, Rapale, and Meconta each have an ophthalmology technician (with an average level of education and training lasting 18 months), and Mossuril does not have any professional [28].

Therefore, these districts have a ratio of eye health professionals per number of inhabitants below that recommended by the World Health Organization (1 Ophthalmic technician per 100,000 inhabitants, 1 optometrist for 250,000 inhabitants, and 1 ophthalmologist per 250,000 inhabitants) [29].

The main cause of VI was RE (87.6%), coinciding with studies from Vietnam [20], Nepal [21], Malaysia [23], and Ghana [22].

However, REs are reaffirmed as the main cause of VI, although the effort made under the WHO Vision 2020 initiative [30], it is still a challenge for the health system of several countries, including Mozambique.

The prevalence of RE was 24.7% (with 14.3% hyperopia and 10.4% myopia), similar to the study carried out in Egypt [31] whose prevalence was 22.8%, but myopia (71%) was predominant in relation to hyperopia (29%). While in Saudi Arabia [7], the prevalence was higher (55.5%), and myopia (53.3%) was more prevalent than hyperopia (2.2%). In Ethiopia [32], the prevalence was lower (9.4%), with myopia having a higher proportion (31.6%) than hyperopia (26.4%).

Therefore, myopia was dominant in other studies, which may be associated with the fact that some studies used an autorefractor without cycloplegic, which overestimates the prevalence of myopia [12], and in others, refraction was performed only in schoolchildren with VA less than 6/6, which may underestimate the prevalence of hyperopia, as it does not always have a low VA.

In a study in Burkina Faso [33], using the same refraction technique as our study, a higher prevalence of hyperopia (4.9%) was found in relation to myopia (0.5%), which was associated with the characteristics of the population in that country (such as low literacy and a non-urban lifestyle). In another study carried out in Poland [34], with cycloplegic retinoscopy, a higher prevalence of hyperopia (38%) than myopia (13%) was found. In addition to the use of cycloplegic, it may be associated with the definition of hyperopia adopted in this study (as S.E of at least +1.00D). In another study carried out in Northern Ireland [35], myopia was lower (2.8%) than hyperopia (26%) in children aged 6–7 years, while in children aged 12–13 years myopia was higher (17.7%) than hyperopia (14.7%). In these studies from Northern Ireland [35] and Poland [34], as well as in our study, the prevalence of myopia tended to increase with age, and the opposite happened with hyperopia, and a positive correlation was found between the prevalence of myopia and age (*p* < 0.001), as well as a negative correlation between hyperopia and age (*p* < 0.001) in Poland [34]. The age groups between 15–17 and 18–20 years old were associated, as a risk factor, to the occurrence of myopia, as well as the 11th and 12th grade, while Malema district was associated, as protective factor, for both myopia and hyperopia. In the study carried out in Egypt [31], residence (urban/rural) was not associated with RE, unlike hours in front of the television or computer, which had a significant association with RE. In Iran [6], myopia was significantly associated with male gender (OR: 2.73, *p* < 0.001) and residence, being higher in students living in urban areas (*p* = 0.019), while in Ireland [13], myopia and hyperopia did not have significant association with gender, residence (rural/urban) and socioeconomic level, but had a significant association with age and ethnicity (*p* < 0.001).

The occurrence of different REs may be associated with various exposure factors among different populations, and myopia is associated with high exposure to electronic device screens (such as smartphones, computers, and tablets) as well as great educational pressure and reduced hours spent outdoors, typical of urban areas and developed countries [36]. However, our study involved schoolchildren living in rural regions of northern Mozambique, which may be associated with a low prevalence of myopia to the detriment of hyperopia.

In Mozambique, education is compulsory and free from the grade 1 to 9, which may suggest that students who are able to continue their studies in the post-compulsory phase, until the 11th and 12th grade, have better socioeconomic conditions; however, greater purchasing power associated with access to technologies and greater educational pressure, resulting in a higher incidence of myopia in this group.

The prevalence of uncorrected refractive error (URE) was 16.5%, higher than that found in studies in Nigeria [37], Ethiopia [38] and Indonesia [39], which was 4.8%, 9.5%, and 12.1%, respectively. The prevalence of URE so much reflects the accessibility of eye health services in the country, and it is clear that it is still a major challenge for the National Health System. This is a public health problem that interferes so much in the quality of life and productivity of the individual, with negative effects on the cognitive and psychosocial development of children, however, it is greatly underestimated, since in many countries there is no really effective screening system for eye problems in children [40,41].

REs can be easily corrected with the use of glasses, contact lenses or refractive surgery [42], and its correction would bring significant improvements in health indicators, since, within students with presenting VI, 53% had normal vision with their best correction. Thus, there is a need for future large-scale studies that identify the barriers to accessing eye health services (consultation, purchase of glasses, medication, surgery) in this portion of the population and subsequent intervention in the short, medium and long term in order to reverse this scenario and combating preventable VI.

It is necessary to implement public policies that support a truly effective, comprehensive and timely screening system, according to evidence-based clinical practice guidelines [43], and invest in eye health professionals training, in order to improve the ratio of professionals per population in each district and guarantee universal access to eye health services.

This study was limited by the impossibility of using cycloplegics in screenings during objective refraction, adopting static refraction with the use of a Keeller Professional Streak Retinoscope and subjective refraction to avoid overestimating the prevalence of myopia. Studies have shown that non-cycloplegic retinoscopy and subjective refraction are clinically accurate and can be applied to screening for refractive error in children, showing good agreement with cycloplegic refraction [44,45]. Furthermore, due to the difficulty of performing repeated measurements among children during the program, due to the limited time of the program, a reliability study using intra- and inter-observer measures was not performed.

As for the factors associated with the occurrence of REs, the present study was not thorough in this approach, and taking into account the relatively high prevalence of REs found (24.7%), there is a need for studies that assess in more detail the risk factors that contribute to the occurrence of REs, as well as protective factors, such as the case of Malema district (in relation to hyperopia and myopia), it is important to investigate what makes this district different from others, since all involved districts were socio-economically similar.

In order to improve eye health indicators in children, it would be important that the program also include children from lower levels of education (pre-school and primary), since the second leading cause of VI in this portion was amblyopia, which is more efficiently treated when detected early, within the sensitive period of development (up to 7 years of age) [46]. The program should include an educational component for teachers and guardians (parents), so that they know the signs and symptoms, and the risk factors associated with the development of REs, since the way in which children are educated, implementing healthy habits such as reducing exposure to electronic device screens, as well as spending more time outdoors, can positively contribute to reducing the incidence of myopia [47]. It is important that parents are aware of the importance of periodic eye examination for early detection and preventing preventable and treatable blindness.

## 5. Conclusions

The prevalence of RE and VI found in this study is relatively high, and most causes of VI are preventable and treatable. The results provide preliminary evidence of the need for greater intervention at the school level as it is a privileged and strategic place to promote eye health. Efforts are needed, especially to bring visual health services to remote and inaccessible areas.

## Figures and Tables

**Table 1:** Socio-demographic characteristics of participants and prevalence of visual impairment.

			Uncorrected Visual Impairment		Presenting Visual Impairment		Best-Corrected Visual Impairment	
Variables	N	%	N	%	N	%	N	%
Age (years)								
12–14	259	45.0	28	10.8	22	8.5	15	5.8
15–17	203	35.3	44	21.7	25	12.3	9	4.4
18–20	113	19.7	33	29.2	15	13.3	5	4.4
Gender								
Male	248	43.1	48	19.4	29	11.7	13	5.2
Female	327	56.9	57	17.4	33	10.1	16	4.9
Grade								
8th	179	31.1	15	8.4	12	6.7	9	5.0
9th	124	21.6	19	15.3	15	12.1	8	6.5
10th	60	10.4	10	16.7	5	8.3	3	5.0
11th	106	18.4	28	26.4	16	15.1	4	3.8
12th	106	18.4	33	31.1	14	13.2	5	4.7
District								
Megovolas	148	25.7	19	12.8	14	9.5	7	4.7
Malema	73	12.7	13	17.8	9	12.3	4	5.5
Rapale	64	11.1	13	20.3	7	10.9	4	6.3
Meconta	227	39.5	48	21.1	26	11.5	10	4.4
Mossuril	63	11	12	21.1	6	9.5	4	6.3
VI category								
Normal			470	81.7	513	89.2	546	95.0
Mild VI			32	5.6	31	5.4	15	2.6
Moderate VI			70	12.2	28	4.9	10	1.7
Severe VI			2	0.3	1	0.2	2	0.3
Blindness			1	0.2	2	0.3	2	0.3
VI laterality								
Normal			470	81.7	513	89.2	546	95.0
Unilateral			16	2.8	16	2.6	19	3.3
Bilateral			89	15.5	46	8.2	10	1.7

CI: confidence interval, VI: vision impairment.

**Table 2:** Prevalence of refractive error and association with socio-demographic characteristics.

Refractive Error									
	Emmetropia	Myopia				Hyperopia			
Variables	% (N)	% (N)	OR (CI95%)	ORa (CI95%)	Power	% (N)	OR (CI95%)	ORa (CI95%)	Power
Age (years)									
12–14	81.9 (212)	3.1 (8)	1	1		15.1 (39)	1	1	
15–17	71.9 (146)	13.3 (27)	4.9 ** (2.2–11.1)	5.1 ** (2.3–11.6)	0.999	14.8 (30)	1.1 (0.7–1.9)	n.s.	0.131
18–20	66.4 (75)	22.1 (25)	8.8 ** (3.8–20.4)	12.5 ** (5.2–30.1)	0.999	11.5 (13)	0.9 (0.5–1.9)	n.s.	0.068
Gender									
Male	74.6 (185)	10.9 (27)	1	1		14.5 (36)	1	1	0.069
Female	75.5 (248)	10.1 (33)	0.9 (0.5–1.6)	n.s.	0.105	14.1 (46)	1.0 (0.6–1.5)	n.s.	
Grade									
8th	84.4 (151)	2.8 (5)	1	1		12.8 (23)	1	1	
9th	77.4 (96)	5.6 (7)	2.2 (0.7–7.1)	n.s.	0.677	16.9 (21)	1.4 (0.8–2.7)	n.s.	0.567
10th	78.3 (47)	8.3 (5)	3.2 (0.9–11.6)	n.s.	0.907	13.3 (8)	1.1 (0.5–2.7)	n.s.	0.088
11th	67.0 (71)	17.9 (19)	8.1 ** (2.9–22.5)	n.s.	0.999	15.1 (16)	1.5 (0.7–3.0)	n.s.	0.566
12th	64.2 (68)	22.6 (24)	10.7 ** (3.9–29.1)	n.s.	0.999	13.2 (14)	1.4 (0.7–2.8)	n.s.	0.144
District ●									
Megovolas	77.0 (114)	6.8 (10)	0.6 (0.3–1.1)	n.s.	0.216	16.2 (24)	1.2 (0.7–2.0)	n.s.	0.224
Malema	82.2 (60)	12.3 (9)	1.1 (0.5–2.3)	0.4 * (0.2–1.0)	0.098	5.5 (4)	0.3 * (0.1–0.9)	0.4 * (0.1–1.0)	0.999
Rapale	75.0 (48)	10.9 (7)	1.1 (0.5–2.5)	n.s.	0.138	14.1 (9)	1.0 (0.5–2.1)	n.s.	0.171
Meconta	74.4 (169)	12.8 (29)	1.5 (1.0–1.1)	n.s.	0.254	12.8 (29)	0.9 (0.5–1.4)	n.s.	0.793
Mossuril	66.7 (42)	7.9 (5)	0.8 (0.3–2.2)	n.s.	0.229	25.4 (16)	2.3 * (1.2–4.2)	2.0 * (1.1–3.8)	0.999
Total	75.3 (433)	10.4 (60)				14.3 (82)			

● The reference group is the rest of the districts; CI: confidence interval, OR: Odds ratio, ORa: adjusted Odds Ratio by socio-demographic variables. Power, Statistic Power for logistic regression. * Statistic signification < 0.05, ** Statistic signification < 0.001.

**Table 3:** Characteristics of refractive errors.

Refractive State	Emmetropia	Myopia			Hyperopia			Astigmatism		
*n* (%)	433 (75.3)	60 (10.4)			82 (14.3)			48 (8.3)		
Severity		mild	moderate	high	mild	moderate	high	WTR ^1^	ATR ^2^	OBL ^3^
*n* (%)		37 (6.4)	22 (3.8)	1 (0.2)	54 (9.4)	27 (4.7)	1 (0.2)	37 (6.4)	4 (0.7)	7 (1.2)

^1^ with-the-rule; ^2^ against-the-rule; ^3^ oblique.

**Table 4:** Causes of visual impairment.

Causes	Uncorrected VI		Presenting VI		Best-Corrected VI	
	N	%	N	%	N	%
Refractive error	76	72.4	33	31.4	0	0.0
Amblyopia	16	15.2	16	15.2	16	15.2
Congenital cataract	4	3.8	4	3.8	4	3.8
Corneal opacity	5	4.8	5	4.8	5	4.8
Toxoplasmosis	1	1.0	1	1.0	1	1.0
Retinal disorders	1	1.0	1	1.0	1	1.0
Other causes	2	1.9	2	1.9	2	1.9
Total	105	100	62	59.0	29	27.6

VI: vision impairment.
